# Stage specific comparative transcriptomic analysis to reveal gene networks regulating iron and zinc content in pearl millet [*Pennisetum glaucum* (L.) R. Br.]

**DOI:** 10.1038/s41598-021-04388-0

**Published:** 2022-01-07

**Authors:** C. Tara Satyavathi, Rukam S. Tomar, Supriya Ambawat, Jasminkumar Kheni, Shital M. Padhiyar, Hiralben Desai, S. B. Bhatt, M. S. Shitap, Ramesh Chand Meena, Tripti Singhal, S. Mukesh Sankar, S. P. Singh, Vikas Khandelwal

**Affiliations:** 1 ICAR-AICRP on Pearl Millet, Agriculture University, Jodhpur, Rajasthan 342 304 India; 2 Department of Biotechnology, Junagadh Agricultural University, Junagadh, Gujarat India; 3 Department of Agricultural Statistics, Junagadh Agricultural University, Junagadh, Gujarat India; 4 Division of Genetics, Indian Agricultural Research Institute, ICAR, New Delhi, India

**Keywords:** Biotechnology, Molecular biology

## Abstract

Pearl millet is an important staple food crop of poor people and excels all other cereals due to its unique features of resilience to adverse climatic conditions. It is rich in micronutrients like iron and zinc and amenable for focused breeding for these micronutrients along with high yield. Hence, this is a key to alleviate malnutrition and ensure nutritional security. This study was conducted to identify and validate candidate genes governing grain iron and zinc content enabling the desired modifications in the genotypes. Transcriptome sequencing using ION S5 Next Generation Sequencer generated 43.5 million sequence reads resulting in 83,721 transcripts with N_50_ of 597 bp and 84.35% of transcripts matched with the pearl millet genome assembly. The genotypes having high iron and zinc showed differential gene expression during different stages. Of which, 155 were up-regulated and 251 were down-regulated while during flowering stage and milking stage 349 and 378 transcripts were differentially expressed, respectively. Gene annotation and GO term showed the presence of transcripts involved in metabolic activities associated with uptake and transport of iron and zinc. Information generated will help in gaining insights into iron and zinc metabolism and develop genotypes with high yield, grain iron and zinc content.

## Introduction

Pearl millet (*Pennisetum glaucum* (L.) R. Br.) is a highly nutritious and multipurpose cereal crop primarily cultivated on more than 27 million ha in the arid and semi-arid tropical regions of Africa and Asia. It is a chief source of dietary energy for the rural people in these areas. It is well adapted to the adverse conditions like drought, high salinity, low pH, low soil fertility and high temperature making it an excellent climate-resilient crop and put forward good security against crop failure in developing countries^1,2^. It is nutritionally better than rice and wheat due to presence of higher quantity of proteins, dietary fibers, iron, zinc, phosphorus, calcium, potassium, vitamin B and essential amino acids and delivers 80–90% of the calories to several millions of poor people across the world^3^. It supplies 19 to 63% of the total iron (Fe) intake and 16 to 56% of the total zinc (Zn) intake from pearl millet based foods in Maharashtra, Gujarat and Rajasthan states of India^4^. In comparison to other cereals and vegetables, it is cheaper and more economical source of Fe and Zn. It has enormous genetic variability for the key mineral elements- iron and zinc when compared to other cereal crops^5^.

Nutritional security is a key challenge for the growing population of the world which is primarily due to micronutrient deficient cereal based diet. Iron and zinc deficiencies are among the two most common and prevalent micronutrient deficiencies (MNDs) leading to ill health, high mortality, lower work productivity, learning disabilities in children and meager national economic development^6,7^. Among the 26 foremost risk aspects of the world, Fe deficiency ranks 9th and Zn deficiency ranks 11th^8^. This is a major issue mainly in developing countries, particularly for infants, adolescent children and pregnant women. In India, approximately 80% of pregnant women, 52% of non-pregnant women and 74% of children of 6–35 months suffer from anaemia caused due to iron deficiency^9^. Zinc deficiency leads to diarrhoea, stunted physical growth and repressed immune system affecting around 50% of the world population^10^. More than three billion people are affected by MNDs of essential minerals and vitamins globally^11^. Breeding and developing crop varieties rich in these elements through biofortification is usually considered as a cost-effective approach to eliminate Fe and Zn deficiencies^12^.

Pearl millet exhibits higher Fe density in comparison to the other major cereals and ‘*iniadi’* pearl millet germplasm is the main basis of breeding lines, improved populations, hybrid parents and composites containing high Zn and Fe densities. Research on pearl millet revealed that it has huge genetic variation for Zn and Fe densities with majorly additive gene action with better parent heterosis. This enormous genetic variation (30–140 mg/kg Fe and 20–90 mg/kg Zn) can be efficiently exploited for developing high yielding cultivars with enhanced Zn and Fe densities^1,2,13,14^. Pearl millet also possesses high bioavailability (absorption) of iron which can fulfill > 50% daily requirement of children or adult males. Approximately 50–100% of the daily allowance of iron is provided by one meal of biofortified high iron variety of pearl millet and proved helpful in eliminating iron deficiency in women, men and children^15^.

Morphological and physiological responses are controlled by numerous genes and significantly prejudiced by the environment. Transcriptomics and metabolomics can prove useful to identify gene function and increase production of important compounds in pearl millet by understanding gene-to-metabolite pathways^16^. Development of next-generation sequencing has provided the opportunity for high-throughput sequencing and used as a valuable tool with vast potential and applications in plant biology including genome sequencing and transcriptome analysis^17^. The next-generation sequencing (NGS) technology is a boon as it overcomes the different inadequacies of microarrays making it most preferable method of transcriptome profiling in recent years.

Transcriptomic studies in pearl millet have been used to reveal the functions of salinity and heat stress-responsive genes such as PgDREB2A, PgNHX1, PgDHN, PgVDAC, Pghsp 16.97^18–23^. A comprehensive transcriptome analysis for drought stress response has also been carried out in pearl millet^24^. The molecular mechanisms underlying salinity tolerance, physiological analyses and a comparative transcriptome analysis were revealed using salinity tolerant (ICMB 01222) and salinity susceptible (ICMB 081) lines under control and salinity conditions and around 11,627 differentially expressed genes (DEGs) were reported in both lines^25^.

In the present experiment, comparative transcriptome profiling of stage-specific spikes from pearl millet with high and low Fe and Zn was carried out to know about the genes that are expressed during developing spike and the transcripts whose expression is associated with Fe and Zn.. This study provides valuable candidate genes that can be genetically targeted for yield along with improvement for Fe and Zn content in pearl millet.

## Material and methods

### Plant material and growth conditions

This experiment was conducted at PC Unit, ICAR-AICRP on Pearl Millet, Agriculture University, Jodhpur during *kharif* 2018. A total of four inbred lines viz. PPMI 953, PPMI 1108, PPMI 627, 5540 B were sown in field conditions following recommended agricultural practices. The four inbred lines were grouped in two categories- high and low, based on Fe and Zn content in the grains of the genotypes as measured by Atomic Absorption Spectrophotometer (AAS). Out of the four inbred lines, the two lines namely PPMI 953 and PPMI 1108 were having high Fe & Zn content and the other two viz., PPMI 627 and 5540 B were having low Fe & Zn content and were considered as control. The samples were collected at three stages i.e., panicle initiation [28 Days After Sowing (DAS)], flowering stage (36 DAS) and milking stage (52 DAS) in 3 replicates for transcriptomic analysis. The samples were named as 1A for the sample collected at panicle initiation from high Fe and Zn containing genotype PPMI 953, 1B for the sample collected at flowering and 1C for the sample collected at milking stage from the genotype PPMI 953. Similarly, the other samples were named as 2A, 2B and 2C for genotype PPMI 1108; 3A, 3B and 3C for genotype PPMI 627 and 4A, 4B and 4C for the genotype 5540 B (Tables 1, 2). The samples were stored in RNAlater (QiaGen) solution in 15 ml tubes under sterilized conditions at 4 °C in a freezer for later use. The transcriptome sequencing and analysis was carried out at Department of Biotechnology, Junagadh Agriculture University, Junagadh, Gujarat.

**Table 1:** Sample details and concentration of Iron (Fe) and Zinc (Zn) in the grains of pearl millet genotypes.

S. No	Sample ID	Genotypes	Description	Fe (ppm)	Zn (ppm)
High Fe and Zn containing Pearl millet Genotype (HFZPG)					
1	1A	PPMI 953	Panicle initiation	103.23	77.53
2	1B	PPMI 953	Flowering stage		
3	1C	PPMI 953	Milking stage		
4	2A	PPMI 1108	Panicle initiation	93.71	56.95
5	2B	PPMI 1108	Flowering stage		
6	2C	PPMI 1108	Milking stage		
			Mean	98.47	67.24
			SD	5.68	11.80
Low Fe and Zn containing Pearl millet Genotype (LFZPG)					
7	3A	PPMI 627	Panicle initiation	50.33	30.51
8	3B	PPMI 627	Flowering stage		
9	3C	PPMI 627	Milking stage		
10	4A	5540 B	Panicle initiation	32.31	26.62
11	4B	5540 B	Flowering stage		
12	4C	5540 B	Milking stage		
			Mean	41.32	28.57
			SD	9.96	2.32
*t-test* significance (between H & L) at *p* < 0.01 *p* value				0.00000024	0.000013

**Table 2:** Reads, de-novo assembly and mapping statistics of 2 high and 2 low-Fe & Zn containing genotypes of pearl millet.

Sr. No	Sample ID	Genotypes	Stages	Total number of raw reads	Total number of high quality reads & percentage after quality control	Percentage of aligned reads with genes of pearl millet de novo assembly
High Fe and Zn containing Pearl millet Genotype (HFZPG)						
1	1A	PPMI 953	Panicle initiation	14,765,667	12,689,248 (84.94%)	85.22
2	1B	PPMI 953	Flowering	13,000,597	11,118,391 (86.06%)	83.92
3	1C	PPMI 953	Milking	10,124,113	7,775,336 (76.6%)	82.39
4	2A	PPMI 1108	Panicle initiation	17,116,512	14,773,081 (86.06%)	84.15
5	2B	PPMI 1108	Flowering	13,456,265	11,534,461 (85.72%)	85.74
6	2C	PPMI 1108	Milking	9,961,928	7,996,499 (80.27%)	82.62
Low Fe and Zn containing Pearl millet Genotype (LFZPG)						
7	3A	PPMI 627	Panicle initiation	14,296,802	12,102,565 (84.65%)	86.71
8	3B	PPMI 627	Flowering	17,410,659	14,959,020 (85.92%)	84.48
9	3C	PPMI 627	Milking	11,035,612	8,60,2071 (77.95%)	83.52
10	4A	5540 B	Panicle initiation	12,865,870	10,878,273 (84.55%)	85.64
11	4B	5540 B	Flowering	20,433,511	15,922,075 (91.44%)	85.13
12	4C	5540 B	Milking	12,289,745	10,872,938 (88.47%)	82.79

### RNA extraction and high-throughput sequencing

Total RNA was extracted from each replicate of the 12 samples (Table 1) using RNeasy Plant Mini Kit (*QiaGen*). The quality of RNA was analyzed on 1.2% agarose gel electrophoresis and concentration was measured using a Qubit RNA HS Assay Kit (Invitrogen) along with the Qubit 2.0 Fluorometer (Invitrogen). mRNA was extracted using Dynabeads mRNA DIRECT Kit (Thermofisher Scientific) from ~ 1 μg of total RNA input. After purification, mRNA was fragmented by RNase enzyme at 37 °C in RNase buffer provided in cDNA library preparation kit *Total RNA*-*Seq Kit v2* (Thermofisher Scientific). These fragmented mRNA were hybridized and ligated with adapters followed by SPRI cleanup (Solid Phase Reversible Immobilization). The fragments were reverse transcribed using random hexamers and superscript II reverse transcriptase (Life Technologies) and further cleaned using Beckman colter agencourt ampure XP SPRI beads. The cDNA library was amplified using PCR for the enrichment of the adapter-ligated fragments. The individual libraries were measured using a Qubit 2.0 Fluorometer and validated for quality in E-Gel 2% Agarose (Invitrogen). Subsequently, these libraries of each sample were diluted up to 100 pM concentration and subjected to emulsion PCR (Ion One Touch 2, Life Technologies). Later, enriched template positive ion sphere particles were loaded on 540 chip and were sequenced in ION S5 next generation sequencer platform (Life technologies).

### Preprocessing of RNA-Seq data and transcriptome profile analysis

Initially, low-quality sequences were removed from the raw reads from all the individual data files. The reads having > 50% bases with low-quality scores and/or > 10% bases unknown (N bases) were removed from each raw data for accuracy of results using CLC genomics tool kit^26^.

### De novo transcriptome assembly, read mapping and sequence annotation

The reads were processed for sequencing adapter removal, further trimmed for quality and ambiguity. The de novo assembly was conducted using default parameters in the CLC Genomics workbench 20.01 (CLC bio, Aarhus, Denmark) to develop contigs/transcripts for all the samples. The transcript reads were further matched to the draft genome of pearl millet^27^ to find out the percentage match. BlastX of assembled transcripts was done with the close relative of pearl millet i.e. *Foxtail millet* protein database with an e-value cut-off of 1E-6, as the protein database of pearl millet was not available. Sequence annotation was carried out with the software BLAST2GO. Based on these results, gene ontology (GO) terms were assigned to the sequences^28^.

### Differential gene expression analysis

Two groups were generated by combining the reads of High Fe and Zn containing Pearl millet Genotype (HFZPG) and Low Fe and Zn containing Pearl millet Genotype (LFZPG). For HFZPG, the reads of sample number 1A, 1B, 1C, 2A, 2B and 2C were combined and for LFZPG the reads of sample number 3A, 3B, 3C, 4A, 4B and 4C were combined. The reads were aligned back to the assembled main assembly to quantify the abundance of transcripts assembled and to determine the number of reads and reads per kilo million (rpkm) corresponding to each transcript. CLC RNA-Seq analysis was used for aligning the reads of each sample onto the assembled transcripts^29^. Assembler provides the annotated transcripts and their annotated length and coverage values for each sample. The resulting files of aligned reads were input in Differential Expression (DE) analysis, a tool for quantifying the abundances of a set of target sequences from sampled subsequence based on a model (GLM model) using the negative binomial distribution^30^. The differential genes were estimated among the different stages of panicle development of low and high Fe and Zn containing genotypes i.e. panicle initiation stage of low Fe and Zn containing genotypes were compared with panicle initiation stage of high Fe and Zn containing genotypes. Similarly, flowering and milking stages were also compared. Gene expression levels were estimated by RPKM and FDR *p* values. Genes with RPKM fold changes > 2 or < − 2, and FDR-corrected *p* values < 0.05 were regarded as Differentially Expressed Genes (DEGs). DEGs transcripts were annotated for pathway analysis and analyzed in MapMan tool for data visualization^31,32^.

### Ethical statement

All the experiments conducted under this study are relevant and according to institutional, national and international guidelines and legislation.

## Results

### Identification of Fe and Zn concentration

The Fe and Zn concentration in the grains of four pearl millet genotypes PPMI 953, PPMI 1108, PPMI 627 and 5540 B was analyzed by atomic absorption spectrophotometer. Fe concentration in high Fe containing genotypes PPMI 953and PPMI 1108 was found to be 103.23 ppm and 93.71 ppm, respectively which was significantly higher than low Fe containing genotype PPMI 627 (50.33 ppm) and 5540 B (32.31 ppm). Similarly, the Zn concentration in PPMI 953, PPMI 1108, PPMI 627 and 5540 B was found to be 77.53 ppm, 56.95 ppm, 30.51 ppm and 26.62 ppm, respectively (Table 1). A significant genetic variation in Fe and Zn concentration was found among high and low Fe genotypes. The iron concentration in the genotype PPMI 953 is sufficient enough to fulfill the desired iron concentration of daily human need^33^. Ample work has been carried to enhance iron and zinc content in pearl millet^34–41^, however little or negligible work has been carried out to understand the mechanism or pathways involved in high Fe and Zn containing genotypes.

### RNA sequencing and assembly

The samples for RNA-sequencing were collected from four pearl millet genotypes (two genotypes for high Fe and Zn while two for low Fe and Zn) at three different stages i.e. panicle initiation stage, flowering stage and milking stage. The samples were collected in three replicates for each stage. The total RNA was isolated from each replicate and were pooled before sequencing to make a total of 12 samples. The sequencing was carried out in Ion S5 next generation sequencing platform with a chemistry of 200 bp. The total number of raw reads varied from approximately 9- 20 million reads for the samples of different stages. The raw reads were further subjected to adaptor trimming and bases quality check. The percentage of high quality (HQ) reads varied from 76.6% to 91.44% and the number of HQ reads varied from ~ 7 to 15 million (Table 2). Mapping of HQ reads with draft pearl millet genome showed 82.39% to 86.71% of reads mapped. Further, the HQ reads of all the 12 samples were subjected to master assembly by combining all the reads of all the samples. A total of 83,721 contigs were generated with an average contig length of 519 bp and N_50_ of 597 bp (Table 3). The complete data has been submitted in National Center for Biotechnology Information (NCBI) with Submission ID:SUB10645683 and BioProject ID:PRJNA779559.

**Table 3:** d*e Novo* assembly statistics of master assembly.

Features	Contig length
N_75_	353 bp
N_50_	597 bp
N_25_	1204 bp
Minimum	200 bp
Maximum	7492 bp
Average	519 bp
Count	83,721
Total	43,459,438 bp

### Differentially expressed genes in high and low Fe and Zn genotypes

**Figure 1:**
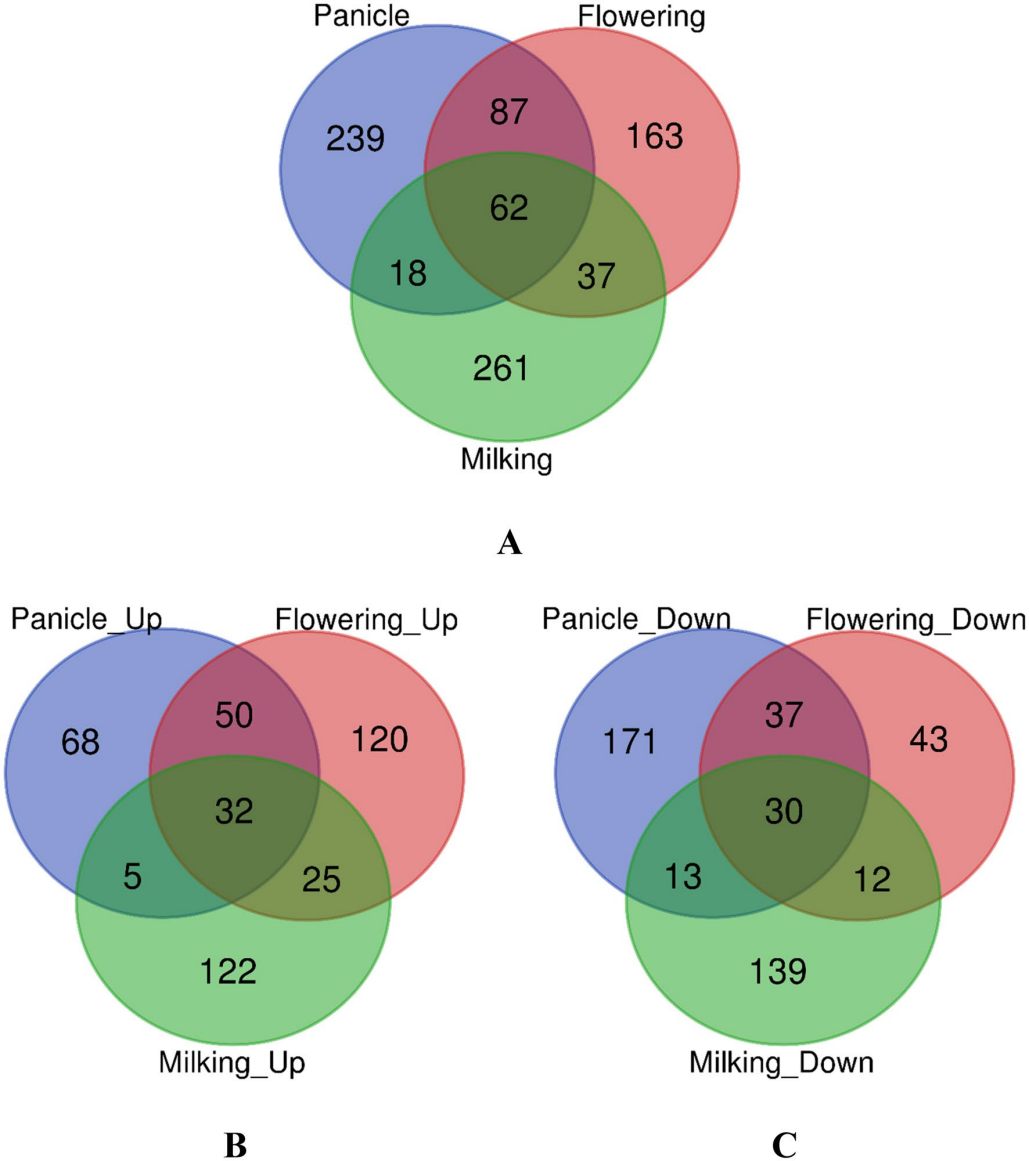
Characterization of Differentially Expressed Genes (DEGs) in pearl millet genotypes during different stages of panicle developmental i.e. Panicle initiation, Flowering and Milking. (**A**) Over-all distribution of differentially expressed genes during different stage, (**B**) Distribution of up-regulated genes among the different stage of panicle development and (**C**) Distribution of down-regulated genes among the different stage of panicle development.

Differentially Expressed Genes (DEGs) were defined as the fold change of the normalized (RPKM) expression values of at least 2 transcripts/contigs in both direction of log2 ratio ≥ 1 and *p* value ≤ 0.05. During panicle initiation stage, a total of 406 transcripts were differentially expressed of which 155 were up-regulated and 251were down-regulated in high Fe and Zn containing genotypes in comparison to low Fe and Zn containing genotypes. During flowering stage, 349 transcripts were differentially expressed of which 227 were up-regulated and 122 were down regulated. Similarly, during milking stage, 184 transcripts were up-regulated and 194 transcripts were down-regulated in HFZPG in comparison to LFZPG (Table 4). The GO terms with their gene description and other functional activities of all the up and down regulated transcripts have been described in Supplementary Table S1 to S6. Among the differentially expressed genes, 62 transcripts were expressed during all the three stages of panicle development, while 239 transcripts were specifically expressed during panicle initiation and 163 and 261 transcripts were specifically expressed during flowering and milking stage, respectively (Fig. 1A). During panicle initiation and milking stage, 18 transcripts were expressed commonly, while during panicle and flowering stage, 87 transcripts were expressed commonly during both the stages.

**Table 4:** Differentially Expressed Genes (DEGs) in pearl millet genotypes during different stages of panicle developmental and in high Fe and Zn containing pearl millet genotypes.

	Stages	Total	Up-regulated	Down-regulated
1	Panicle Initiation	406	155	251
2	Flowering	349	227	122
3	Milking	378	184	194
	Total	1,133	566	567

When the up-regulated genes were characterized, it was found that there were 32 transcripts which were up-regulated during all the three stages of panicle development. However, 68 transcripts up-regulated only during panicle initiation stage and 120 and 122 transcripts up-regulated only during flowering and milking stages (Fig. 1B). While among the down-regulated genes, 30 transcripts were down-regulated during all the three stages of panicle development (Fig. 1C).

### Functional annotation of DEGs

**Figure 2:**
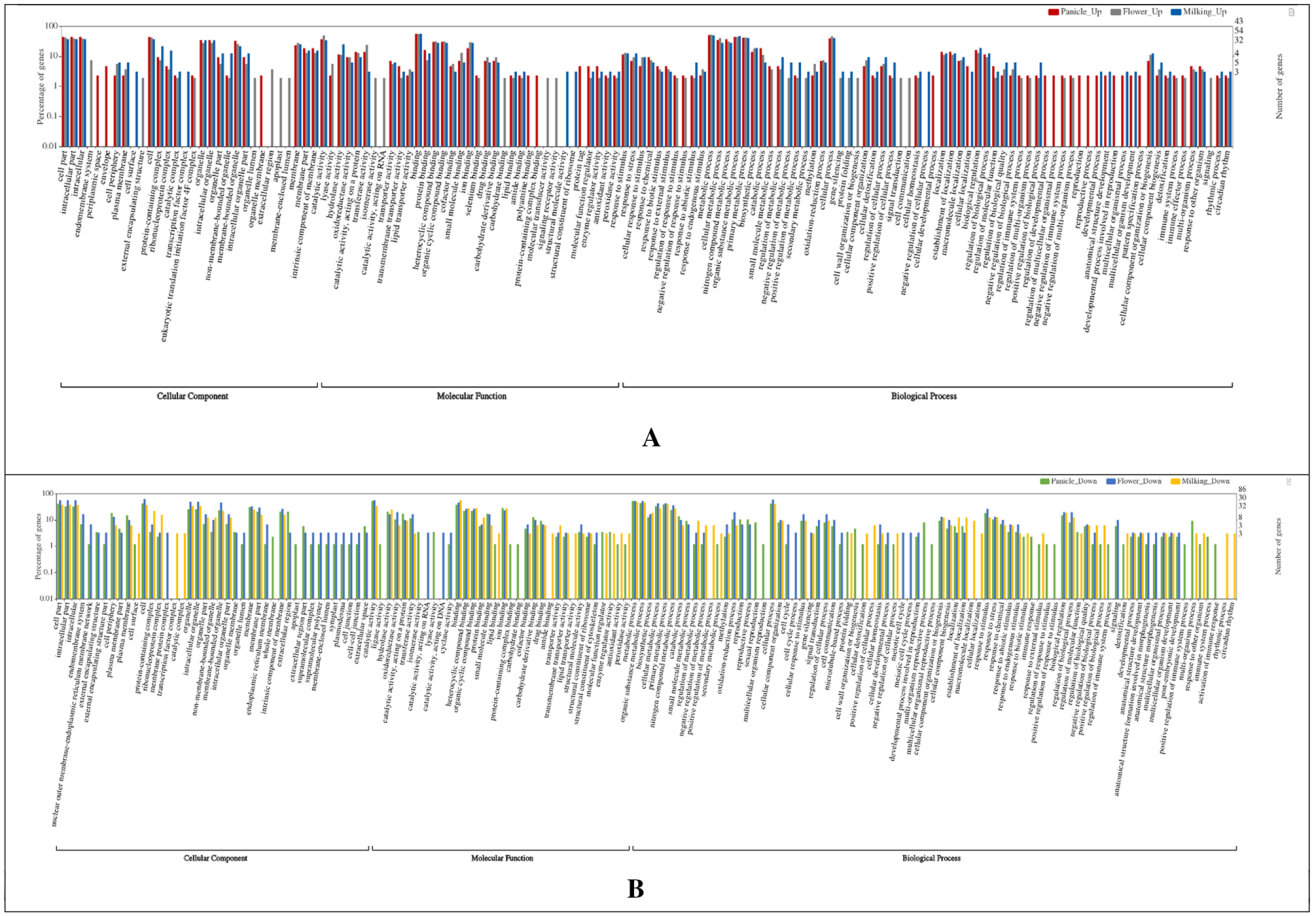
GO enrichment analysis of DEGs was classified into (**A**) up-regulated cellular components, molecular function and biological process; (**B**) down-regulated cellular components, molecular function and biological process.

**Figure 3:**
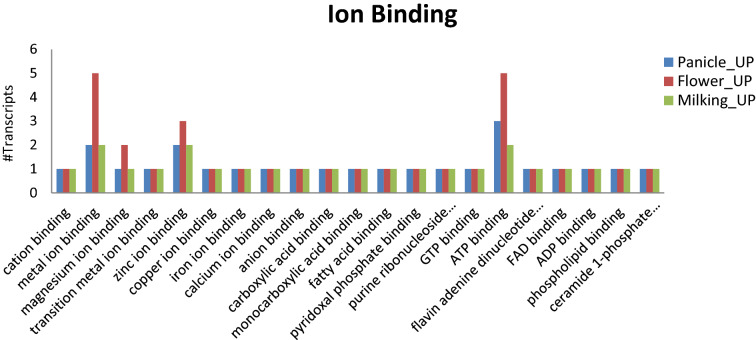
Classification of ion binding component of molecular function which were up-regulated during the three different stages of panicle development i.e. panicle initiation, flowering stage and milking stage.

To identify the putative function, pearl millet’s differentially expressed genes were compared against the *Foxtail millet* protein database using BLASTx search. Only 1,133 transcripts of HFZPG which includes 566 up-regulated and 567 down-regulated were used for functional annotation and assigning GO-based classification (Fig. 2). Cellular components (CC) GO terms i.e. plasma membrane, intracellular, cell periphery, protein-containing complex, transcription factor complex, membrane-bounded organelle, organelle membrane, cytoplasm, membrane, cytosol, extracellular region, apoplast, membrane, etc. were found to be enriched, indicating their participation in the uptake of micronutrients. Biological process (BP) GO terms likewise oxidation–reduction process, regulation of transcript, macromolecule localization, establishment of localization, metabolic process, organic substance metabolic process, biosynthetic process, small molecule metabolic process etc*.* were associated with up-regulated genes. The presence of nucleoside-triphosphate phosphatase as up regulated enzyme has been reported to be involved in different metal ions accumulating metabolic process^42^. Molecular functions (MF) GO terms like ion binding, small molecule binding, amide binding, lipid binding, transporter activity, transferase activity, transmembrane transporter activity, etc. were highly enriched in up-regulated genes. The further classification of ion binding components of MF indicated up-regulation of metal binding, zinc ion binding, iron ion binding, calcium ion binding etc. which may have contributed significantly in the uptake and transport of iron, zinc and other metals (Fig. 3). Fe was involved in chlorophyll synthesis, contributing to the maintenance of chloroplast structure and its function^43^. The presence of metal ion binding, iron ion binding, zinc ion binding and calcium ion binding in HFZPG genotypes indicates their role in accumulation of Fe and Zn in pearl millet genotypes (Fig. 3 and Supplementary Table S7).

### Pathway analysis

**Figure 4:**
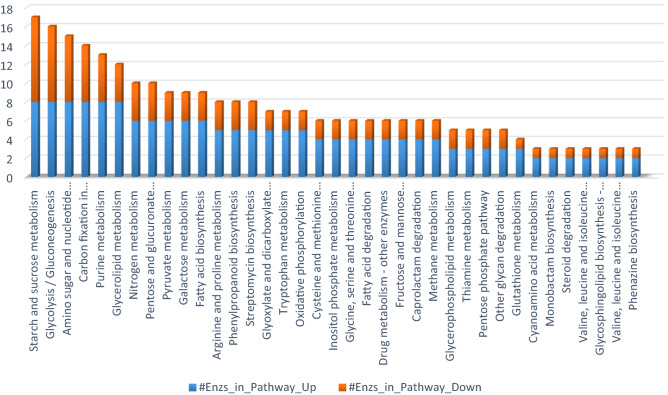
Different metabolic pathways and associated genes encoding enzymes that participated in different metabolic pathways.

**Figure 5:**
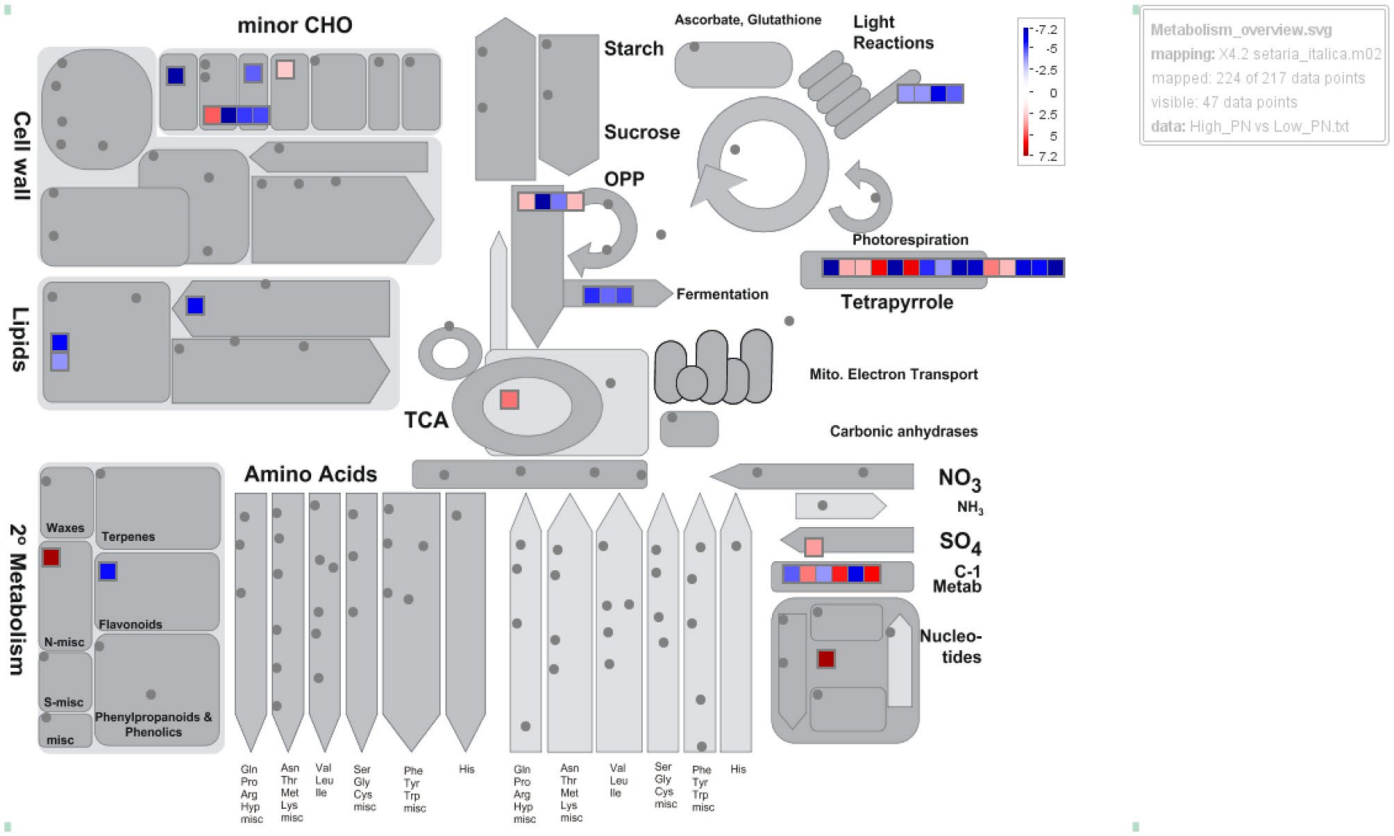
A representation of transcript abundance from the high Fe genotype milking stage versus low Fe genotype milking stage experiment displaying differential gene expression involved in metabolism. (The logarithmic colour scale bar ranges from − 7.2 to + 7.2 (dark red, representing a threefold up‐regulated genes.

Kyto Encyclopedia of Genes and Genomes (KEGG) pathways^44–46^ were analyzed to find out the differences in metabolic processes between HFZPG and LFZPG genotypes (Fig. 4). Pathway results infer that in HFZPG, starch/sucrose metabolism, glycolysis/gluconeogenesis, amino sugar and nucleotide sugar metabolism, carbon fixation in photosynthetic organisms, purine metabolism and glycerolipid metabolism pathways were highly enriched.. MapMan pathway analysis at milking stage of high Fe genotypes as depicted in Fig. 4, indicates up-regulated and down regulated genes in different locations of cell and their level of expression in red to dark blue color range. Genes involved in photorespiration process and carbon metabolism were found to be more up and down regulated (Fig. 5).

### DEGs involved in transportation and uptakes of mineral in HFZPG

Transportation and accumulation of minerals (Zn, Fe, Cu, Ca, Cu, Cd etc.) by plants from soil involves many complex regulatory processes directed by group of genes responsible for enhancing mineral uptake by the roots and their transportation to the most suitable place of accumulation. Different types of protein families involved in transportation of minerals from root/shoot to the other parts of the plant have been identified. In the present study, different protein families have been identified which are involved in different processes of mineral uptake at various stages of panicle development. During different stages of panicle development i.e. panicle initiation, flowering and milking different protein families responsible of transportation and uptake of minerals were either up-regulated or down-regulated in the HFZPG (Table 5 and Supplementary Table S7 and S8). A total of 21 protein families were identified to have function associated with minerals uptake in pearl millet. Among these 21 protein families, all the identified protein family were up-regulated during different stages of panicle development except for ABC transporter family protein and ZC3H15 family which were down regulated during milking stage and flavanone 3-dioxygenase 2 which was down regulated during panicle initiation stage. ABC transporter family proteins having functions like ATP binding, ATPase activity and transmembrane movement of substances, were found to be up-regulated during panicle initiation stage (↑panicle initiation) and down regulated during milking stage (↓milking stage). This indicates that the mineral transport process is active during panicle initiation stage and further deactivated during flowering and milking stage. Three transcripts related to multicopper oxidase family were up-regulated during panicle initiation and were responsible for copper ion binding, iron ion transport and iron ion homeostasis. There were few protein families which were up-regulated only during panicle initiation viz*.,* ABC transporter family proteins, Multicopper oxidase family, cytochrome P450 family and Peroxidase family, while one protein family was up-regulated only during flowering stage i.e. terpene synthase family, while 7 protein families were up-regulated only during milking stage. Protein families like catalase family, major facilitator superfamily, E3 ubiquitin-protein ligase RZFP34 and ZC3H15/TMA46 family were up-regulated during all the three stages of investigation.

**Table 5:** List of transcripts along with their protein family having a functional association with minerals uptake in high Fe and Zn containing pearl millet genotypes (HFZPG).

	Transcript ID	Protein family	Gene description	Predicted functions	Stage of panicle development when expressed
1	4B(single)_trimmed_contig_73886	ABC transporter family proteins	ABC transporter F family member 1	ATP binding; ATPase activity, coupled to transmembrane movement of substances	↑Panicle Initiation ↓ Milking stage
2	4B(single)_trimmed_contig_5720 4B(single)_trimmed_contig_372084B(single)_trimmed_contig_16869	Multicopper oxidase family	L-ascorbate oxidase homolog; laccase-15	Ferroxidase activity; copper ion binding; plasma membrane; iron ion transport; lignin catabolic process; apoplast; hydroquinone: oxygen oxidoreductase activity; iron ion homeostasis; obsolete oxidation–reduction process	Panicle Initiation
3	4B(single)_trimmed_contig_7271	Catalase family	Catalase isozyme	Catalase activity; heme binding; metal ion binding	↑Panicle Initiation ↑Flowering stage ↑Milking stage
4	4B(single)_trimmed_contig_55418 4B(single)_trimmed_contig_55418	Flavoprotein pyridine nucleotide cytochrome reductase family	Cytochrome b5	Cytoplasm; integral component of membrane; heme binding; intracellular membrane-bounded organelle; metal ion binding	↑Panicle Initiation ↑Flowering stage
5	4B(single)_trimmed_contig_5470 4B(single)_trimmed_contig_1419	Heavy metal‐associated isoprenylated plant protein family	Heavy metal-associated isoprenylated plant protein	Metal ion transport; metal ion binding	↑Panicle Initiation ↑Flowering stage
6	4B(single)_trimmed_contig_66144	Major facilitator superfamily	SPX domain-containing membrane protein Os02g45520	Reverse transcriptase zinc-binding domain	↑Panicle Initiation ↑Flowering stage ↑Milking Stage
7	4B(single)_trimmed_contig_81256 4B(single)_trimmed_contig_58468	Cytochrome P450 family	Cytochrome P450 87A3	Heme binding; iron ion binding; monooxygenase activity; oxidoreductase activity	↑Panicle Initiation
8	4B(single)_trimmed_contig_48605	Peroxidase family	Peroxidase 31	Heme binding; metal ion binding peroxidase activity; extracellular region; response to oxidative stress; obsolete oxidation–reduction process; cellular oxidant detoxification	↑Panicle Initiation
9	4B(single)_trimmed_contig_10249 4B(single)_trimmed_contig_8196		E3 ubiquitin-protein ligase RZFP34	Zinc ion binding; ubiquitin protein ligase activity	↑Panicle Initiation ↑Flowering stage ↑Milking Stage
10	4B(single)_trimmed_contig_9268 4B(single)_trimmed_contig_72417 4B(single)_trimmed_contig_12423 4B(single)_trimmed_contig_16541	ZC3H15/TMA46 family	Zinc finger CCCH domain-containing protein 15 homolog	Metal ion binding	↑Panicle Initiation ↑Flowering stage ↓ Milking stage
11	4B(single)_trimmed_contig_11940 4B(single)_trimmed_contig_50921	Mitogen-activated kinase	Mitogen-activated protein kinase kinase kinase 3 isoform X2	Metal ion transportation; Signal transducer, downstream of receptor, with serine/threonine kinase activity	↑Flowering stage ↑Milking Stage
12	4B(single)_trimmed_contig_2313	Zinc-containing alcohol dehydrogenase family	Probable cinnamyl alcohol dehydrogenase 8D	Zinc ion binding; lignin biosynthetic process; cinnamyl-alcohol dehydrogenase activity; obsolete oxidation–reduction process	↑Flowering stage ↑Milking Stage
13	4B(single)_trimmed_contig_75011	Terpene synthase family	Alpha-humulene synthase	Lyase activity; metal ion binding; magnesium ion binding	↑Flowering stage
14	4B(single)_trimmed_contig_11410	Ferredoxin–NADP reductase type 1 family	Ferredoxin	Electron transfer activity; chloroplast; electron transport chain; metal ion binding; iron binding	↑Milking Stage
15	4B(single)_trimmed_contig_59340 4B(single)_trimmed_contig_42031 4B(single)_trimmed_contig_71669	YSL (TC 2.A.67.2) family	Probable metal-nicotianamine transporter YSL7	Oligopeptide transporter; May be involved in the transport of nicotianamine-chelated metals	↑Milking Stage
16	4B(single)_trimmed_contig_19764	Glycosyl hydrolase 13 family	Alpha-amylase type A isozyme	alpha-amylase activity; calcium ion binding; carbohydrate metabolic process; alpha-amylase activity	↑Milking Stage
17	4B(single)_trimmed_contig_74989	Zinc finger transcription factor family	Zinc finger CCCH domain-containing protein 33	Metal ion binding; Transcription factor activity, sequence-specific DNA binding; transcription regulatory region DNA binding; Stress response	↑Milking Stage
18	4B(single)_trimmed_contig_68815	CONSTANS family	Zinc finger protein CONSTANS-LIKE 3	Zinc ion binding; DNA binding; DNA-binding transcription factor activity; identical protein binding	↑Milking Stage
19	4B(single)_trimmed_contig_37316	Multicopper oxidase family	Putative laccase-9	Ferroxidase activity; copper ion binding; plasma membrane; iron ion transport; lignin catabolic process; apoplast; hydroquinone: oxygen oxidoreductase activity; iron ion homeostasis; obsolete oxidation–reduction process	↑Milking Stage
20	4B(single)_trimmed_contig_65409	Serine-threonine kinase family	Family of serine hydrolases 3-like	Metal ion binding	↑Milking Stage
21	4B(single)_trimmed_contig_6082	Iron/ascorbate-dependent oxidoreductase family	flavanone 3-dioxygenase 2	Metal ion binding; dioxygenase activity; obsolete oxidation–reduction process	↓Panicle Initiation

### Genes related to Fe and Zn biosynthesis in HFZPG

In plants, for translocation of minerals from one part to another part, various kind of accumulators have been identified. Transport of Fe and Zn from soil to root and then to shoot is affected by many factors like pH, water availability, organic substances, which may affect transport of Fe and Zn to its target destinations. Many studies have been carried out on accumulator which may play role in mineral transport and also in stress tolerance response (STR)^47^. Functional annotation of high Fe and Zn containing genotypes with foxtail millet database revealed transcripts belonging to different families of genes to be involved in higher Fe and Zn accumulation (Supplementary Table S7 and S8). The functional annotation of the DEG’s indicated few transcripts to be belonging to the families having function related to metal binding and metal ion transport. The protein families identified in the high Fe and Zn containing genotypes having function specifically related to Fe and Zn were multicopper oxidase family, major facilitator superfamily, cytochrome P450 family, zinc-containing alcohol dehydrogenase family, ferredoxin–NADP reductase type 1 family and CONSTANS family (Table 5).

## Discussion

Micronutrient malnutrition due to iron and zinc deficiencies is a serious public health problem in developing countries. Globally malnutrition has been in the prime focus of researchers across the world. The development of cereal crops rich in micronutrients is one of the major approaches to overcome this problem. In India alone, about 74% of children and 80% of the pregnant women suffer from iron and zinc deficiency. At present, knowledge of the genes involved in transportation and accumulation of Fe and Zn in the seeds of pearl millet is not known and hence a study on genes responsible for Fe and Zn has become important. Various strategies for iron uptake, iron transport and iron storage in many agricultural crops mainly in rice, wheat, common bean, maize reported earlier by many researchers. Movement of iron from soil to its destination place is a complex mechanism and require transportation system which is performed by different genes that regulate the mineral uptake^48^.

Transcriptome analysis using Next Generation Sequencing (NGS) technologies is a robust and efficient method for exploring the pattern of gene expression in host. Transcriptome sequencing or RNA-seq surpasses cloning and is helpful in exploring the complexity of transcriptome by allowing RNA analysis through cDNA sequencing at massive scale^49^. It has the potential to identify important secondary metabolic pathways, various transcripts associated with diseases and helpful for discovering novel genes^50^ and developing molecular markers^51^. RNA sequencing (RNA-Seq) is widely used among the different transcriptome analysis methods as it can efficiently detect unknown genes and novel transcripts and has much potential to study gene expression and their regulating pathways^52^. This approach has been successfully applied in many important crops, including sesame^53^, chickpea^54^, finger millet^55^, foxtail millet^56^, peanut^57^, maize^58^ and cotton^59,60^.

In this study, we used transcriptome sequencing for identification of transcripts involved in Fe and Zn accumulation in the pearl millet genotypes. Two sets of pearl millet genotypes having high and low Fe and Zn in their grains were taken. The samples were collected at three stages of spike development i.e. panicle initiation, flowering stage and milking stage. mRNA was isolated and was subjected to sequencing using Ion S5 next generation sequencer. The analysis of the transcripts was carried out individually for each stage and later combining all the transcripts into two group i.e. high Fe and Zn containing pearl millet genotype (HFZPG) and low Fe and Zn containing pearl millet genotype (LFZPG).

The pearl millet developing spikes transcriptome sequence generated 43 million sequence reads resulting in 83,721 transcripts with N_50_ of 597 bp. When matched with the pearl millet de novo assembly, 84.35% of transcripts matched with the genome assembly. Further, when the differentially expressed gene were analyzed, it was found that the maximum number of genes were differentially expressed during the panicle initiation stage of panicle development indicating that the accumulation and transportation of Fe and Zn might be taking place during the panicle initiation stage of panicle development. The group of genotypes having high Fe and Zn showed that during panicle initiation stage a total of 406 transcripts were differentially expressed of which 155 were up-regulated and 251were down-regulated while during flowering stage and milking stage 349 and 378 transcripts were differentially expressed, respectively. The gene annotation and GO term assigning of up-regulated genes showed the presence of transcripts involved in metal ion binding, ATP binding, DNA binding, heme binding, iron ion binding, zinc ion binding, catalytic activity, transferase activity, transferring acyl groups, oxidoreductase activity, peroxidase activity, calcium ion binding, copper ion binding, nutrient reservoir activity, etc. indicating their role in uptake and transport of iron, zinc and other metals. The genes like multicopper oxidase family, major facilitator superfamily, cytochrome P450 family, Zinc-containing alcohol dehydrogenase family, ferredoxin–NADP reductase type 1 family and CONSTANS family having activities related to iron and zinc accumulation were also found to be up-regulated in the transcripts of high Fe and Zn containing genotypes.

The iron we consume from plant-based foods undergoes several processing steps with the help of many plant proteins before it forms the final product in form metal cofactor or in storage form^61^. Enzymes such as proton ATPases, ferric reductases intervene with the solubilization of iron hydroxides in the soil. Further, Fe is transported from the apoplast to the symplast and numerous transporters are needed to distribute it within the plant via xylem and phloem. Specific transporter proteins are required for its transportation across intracellular membranes and biosynthetic enzymes helps in integrating it into heme or iron-sulfur clusters. Later, iron is stored in ferritin in the plastids or in the vacuoles. Hence, any of these proteins/ genes can be utilized as biofortification targets and iron uptake entails many genes^62,63^. But, only a few them have been identified as biofortification targets. Plants possess two main approaches for iron uptake –chelate based approach in grasses and reductive approach in non-grasses. Iron concentration was observed to increase by 1.7-fold in rice leaves while it was 1.1-fold in grains due to over expression of IRT1, a divalent metal transporter which is central to reductive iron uptake^64^. Thus, iron accumulates in the vegetative tissues in the absence of extra sink capacity in seeds. Infact, iron concentration increased in the endosperm up to fourfold in polished rice when IRT1 is overexpressed along with PvFER1^65^. Similarly, collective over expression of Arabidopsis IRT1 and FER1 leads to a 5.5-fold in cassava^66^. During reductive iron uptake mechanism, small molecules such as coumarin-derivatives are secreted in some species while flavins in others^63^, but till now it has not been confirmed if biosynthesis genes may be used for biofortification. Derivatives of NA-mugineic acid and deoxymugineic acid (DMA) are secreted in the rhizosphere in grasses and cereals and chelate Fe^3+^. The Fe-chelator complexes are transported into the cell by YSL15 in rice and YS1 in maize^67^. But, overexpression of IDS3 (Iron Deficiency Specific Clone 3, encoding 2’-deoxymugineic-acid 2’-dioxygenase) and YSL15 by increasing DMA resulted into modest increases in iron concentration in rice seeds^68,69^.

## Conclusion

We successfully used transcriptome sequencing for identification of transcripts involved in Fe and Zn accumulation in the pearl millet genotypes and it is the first report for identification of metabolic pathways involved for Fe and Zn content in pearl millet. This will be further useful in developing hybrids/varieties rich in Fe and Zn content in pearl millet which will aid in mitigating hidden hunger and enhance nutritional security.

## Supplementary Information

Supplementary Tables.

Supplementary Tables.
